# Basal Cell Carcinoma: A Single-Center Experience

**DOI:** 10.5402/2012/246542

**Published:** 2012-12-22

**Authors:** Ozan Luay Abbas, Huseyin Borman

**Affiliations:** Department of Plastic, Reconstructive and Aesthetic Surgery, Baskent University Faculty of Medicine, 40000 Ankara, Turkey

## Abstract

*Background*. Basal cell carcinoma comprises the vast majority of skin cancers. It predominantly affects fair-skinned individuals, and its incidence is rising rapidly. Etiology may be multifactorial, but sun exposure appears to play a critical role. When detected early, the prognosis is excellent. Thus appropriate diagnosis, treatment, and surveillance are of utmost importance. *Methods*. From January 1994 to May 2012, 518 basal cell carcinomas were excised in our clinic. Data were collected retrospectively. *Results*. During 18-year period, 518 BCCs were excised from 486 patients. Most of the patients were males with a median age of 65.6 years. Most of the basal cell carcinomas were located in the head region. Nodular histological subtype dominated our series. Six percent of the excised lesions required reexcision because of involved margins. Our recurrence rate was 6.94% with the nose and the periauricular and periocular regions being the most common sites of occurrence. *Conclusion*. Although there is relatively low attributable mortality, the morbidity and cost of treatment are significant. A large body of information serves as a foundation for oncologic principles, diagnosis methods, surgical excisions, follow-up protocols, and reconstructive methodologies that are currently in use. Surgical ablation remains the mainstay of treatment.

## 1. Introduction

Basal cell carcinoma (BCC) is a malignant epithelial neoplasm that originates from the pluripotential cells in the epidermis and hair follicles [1]. It is the most common skin cancer seen in human population [2]. It is often slow growing and may take years to enlarge significantly [3], but it can cause extensive local tissue destruction and slow death if inadequately treated or left untreated. The mortality rates associated with this cancer are low. However, it causes considerable functional and cosmetic deformity and cost of treatment is significant. In this study, we aimed to share our experience in the management of these tumors. 

## 2. Methods

Our BCC database was reviewed between 1994 and 2012. Variables collected by operating surgeon were the patient's age, sex, tumor site, size, histologic subtype, surgical margin of excision, multiplicity of lesions, presence of involved margins, recurrence during followup, and the presence of metastasis.

All of the pathology specimens were examined and reported by the Department of Pathology at our center. 

## 3. Results

### 3.1. Age and Sex

**Figure 1:**
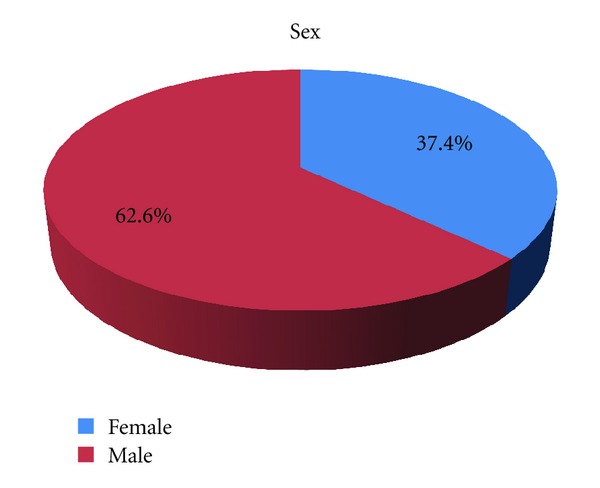
Gender percentages in BCC patients.

From January 1994 to May 2012, 518 BCCs were excised from 486 patients. The median age was 65.6 (range of 20 to 93 years), 182 patients were women (37.4%), and 304 were men (62.55%) (Figure 1).

### 3.2. Anatomical Site

**Figure 2:**
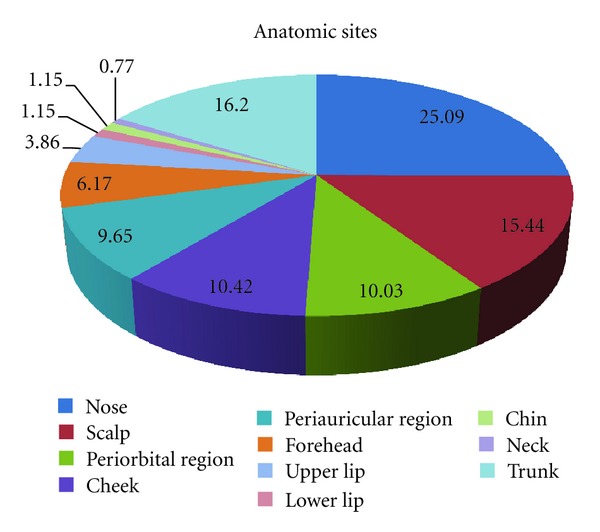
Anatomic distribution of the lesions.

Most basal cell carcinomas were located on the head region (83.8%). Anatomical distributions were as follows: the nose (25.09%), scalp (15.44%), periorbital region (10.03%), cheek (10.42%), periauricular area (9.65%), forehead (6.17%), upper lip (3.86%), lower lip (1.15%), chin (1.15%), and neck (0.77%) (Figure 2). 

### 3.3. Diameter of Lesion

**Figure 3:**
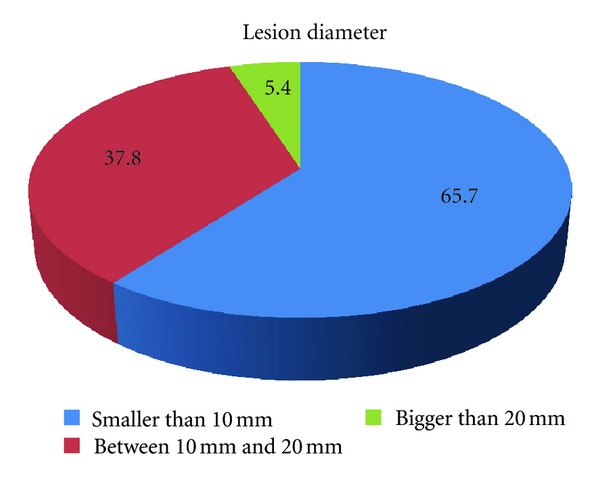
Lesion diameter.

294 lesions were smaller than 10 mm (56.7%), 196 lesions were between 10 and 20 mm (37.8%), and 28 lesions were bigger than 20 mm (5.4%) (Figure 3).

### 3.4. Histologic Subtypes

**Figure 4:**
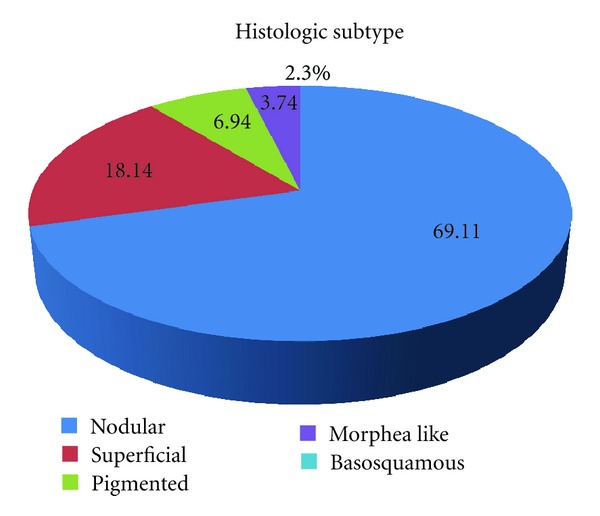
Histologic subtypes.

Among 518 diagnosed BCCs, 358 were nodular (69.11%), 94 were superficial (18.14%), 36 were pigmented (6.94), 18 were morphea like (3.74%), and 12 were basosquamous (2.3%) (Figure 4).

### 3.5. Margin of Excision

For lesions smaller than 1 cm in diameter we used a 3 mm margin for excision. We increased the excision margin 1 mm for every increase of 1 cm in diameter. We used a safety margin of 4 mm for morpheaform BCC and 5 mm for recurrent BCC.

### 3.6. Presence of Multiple Lesions

Only 18 patients (3.7 percent) had more than one lesion excised at the same operation.

### 3.7. Presence of Involved Margins

12 BCCs (2.3%) required reexcision because of involved margins.

### 3.8. Recurrent Lesions

During followup we observed recurrence in 16 cases (3.08%). All occured during the first four years. 8 were in the nose, 4 were in the periauricular area, and 4 were in the periorbital area. 

## 4. Discussion

The majority of BCCs occur in men, and the ratio of male to female in our series is 1.6 : 1. The higher incidence in men is probably due to increased recreational and occupational exposure to the sun. However, the incidence in women is increasing because of changing fashions in lifestyle. 

The likelihood of developing BCC increases with age, and it is rarely found in patients younger than 40 years. The mean age of our patients is 65.6. The damaging effects of the sun begin at an early age. The results may not appear for 20–30 years. 

The exact cause of BCC is unknown. Several factors are believed to predispose the patient to basal cell carcinoma. Exposure to sunlight is the most frequent association. Cumulative exposure to sunlight over years is necessary for tumor development [4]. There are three types of UV radiation: UVA (320–400 nm), UVB (290–320 nm), and UVC (200–280 nm). UVB rays are the most carcinogenic, triggering skin cancer via photochemical damage to DNA, injury to DNA repair mechanisms, and partial suppression of cell-mediated immunity [5]. Patients often have a history of chronic sun exposure. Those at high risk for developing basal cell carcinoma are Fitzpatrick skin types I and II [6]. Because of the climate in our region, most of our patients had a history of chronic sun exposure. 

Ionizing radiation exposure may generate BCC via DNA damage. The minimum reported radiation dosage for inducing skin cancer is 450 rads [7].

 A modest increase in the lifetime risk of basal cell carcinoma has been noted in chronically immunosuppressed patients, such as recipients of organ or stem cell transplants. Immunosuppression alters the immune surveillance mechanism that destroys potentially malignant cells [8]. Six of our patients were immunosuppressed after renal transplantation.

The distribution of basal cell carcinoma across the body varies. Most of these carcinomas occur on sun exposed areas [9]. In our series, 84 percent of basal cell carcinomas are found on head and 16 percent are found on trunk and extremities. The most common site for occurrence is the nose (25.09%)

BCCs can be divided into several subtypes: superficial, nodular, pigmented, morphea like, and basosquamous.

**Figure 5:**
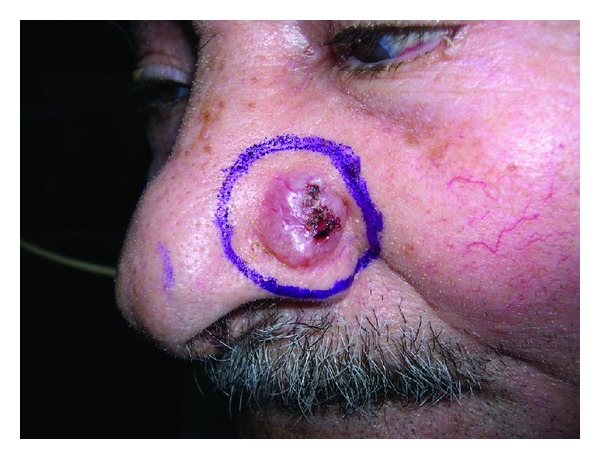
Nodular BCC presents as well-defined translucent pearly nodule that is either round or oval with rolled border and occasional ulceration. Telangiectasias are commonly seen coursing through the lesion.

Nodular BCC is the most common type. It represents 69.11% of our series. Clinically it presents as well defined translucent pearly nodule that is either round or oval with rolled border and occasional ulceration. Telangiectasias are commonly seen coursing through the lesion (Figure 5). Most tumors of this kind are observed on the face.

**Figure 6:**
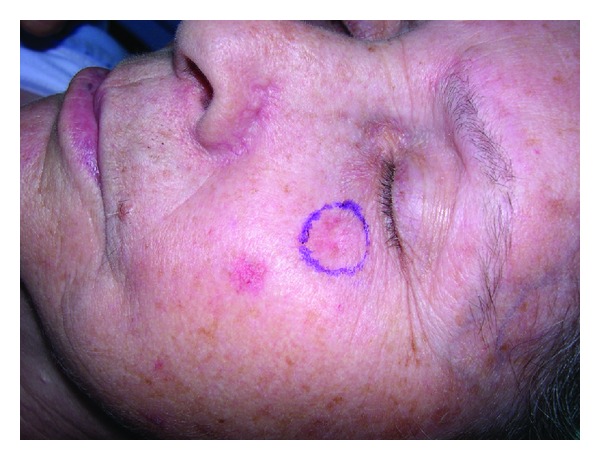
Superficial BCC presents as slightly elevated plaque or discrete macule that may be scaly.

Superficial BCC is the second most common subtype in our series (18%). It presents as slightly elevated plaque or discrete macule that may be scaly [10] (Figure 6), most often developing on the upper trunk or shoulders. Superficial BCC had the lowest percentage of positive margins after excision (3.6%) [11].None of our recurrent BCCs were superficial type.

**Figure 7:**
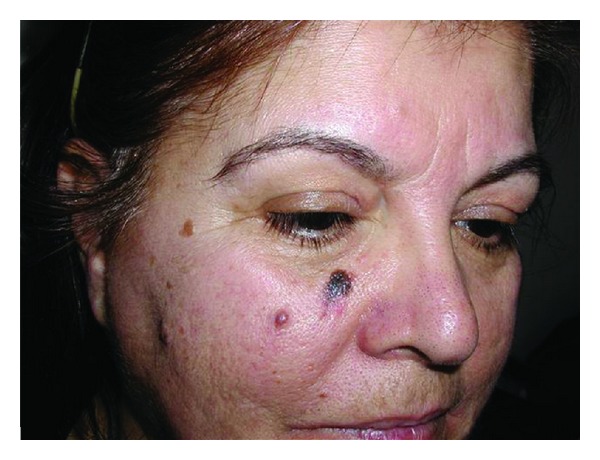
Pigmented BCC.

Pigmented BCC is a rare variant (6.9%). It ranges from brown to blue black and can be mistaken for melanoma (Figure 7). Telangiectasis that are typical of a nodular basal cell carcinoma can be observed. This aids clinically in differentiating this tumor from a melanoma.

**Figure 8:**
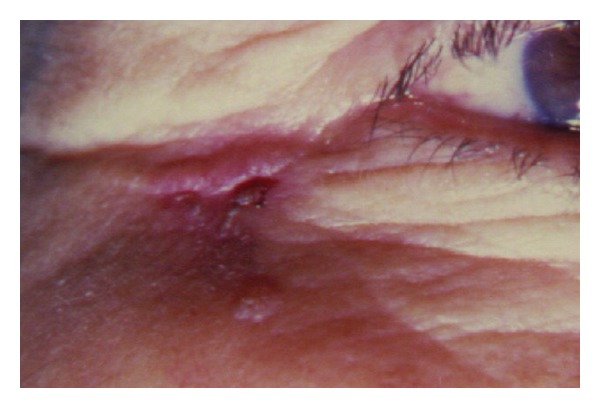
Morpheaform BCC.

Morphea-like BCC is an aggressive rare variant accounting for 3.7% of all our BCCs. It presents as firm plaques that is yellow or white with ill-defined border (Figure 8).Tumor cells induce a proliferation of fibroblasts within the dermis and an increased collagen deposition (sclerosis) that clinically resembles a scar. The extent of the tumor is usually not apparent on clinical examination. Morpheaform BCCs had the highest percentage of positive margins after excision. Thirty-three percent of our morpheaform BCCs had involved margins after excision. Mohs micrographic surgery is valuable in the management of these lesions.

Basosquamous carcinomas have both basal and squamous cell differentiations. They have a higher growth rate as well as higher metastatic potential than do other BCCs. It represents 2.3% of all our series.

Regardless of the appearance of the lesion, we perform a histologic confirmation and typing. The histologic characteristics influence clinical behavior, recurrence, and metastatic potential.

Shave biopsy with a scalpel is a simple method removing the epidermis and a portion of the dermis. Since this tumor arises from the basal layer of the epidermis, shave biopsy will provide sufficient material for histological diagnosis and classification. Approximately 75.9% accuracy rate has been found with shave biopsy [12].We do not prefer this type of biopsy in pigmented BCCs that are difficult to differentiate from the melanomas. 

Punch biopsy garners a full-thickness specimen. The punched out defect may be sutured or may heal secondarily. The accuracy rate with punch biopsy is 80.7% [12]. We only prefer using this type of biopsy when a large lesion of uncertain diagnosis exists. 

Excisional biopsy is the biopsy type that we usually prefer. We advise excisional biopsy for small lesions that enable primary closure afterwards that does not cause distortion of the environmental tissues. Otherwise, an incisional biopsy may be done before the definitive treatment.

Once the pathologic diagnosis of BCC is confirmed, the next step is to plan for tumor eradication by correlating tumor characteristics with patient's age, skin history, medical history, social history, and cosmetic expectations. Treatment options include standard surgical excision, Mohs micrographic surgery, nonsurgical ablation, and topical chemotherapy. 

Surgical excision is the preferred method in our center. We generally perform excision under local anesthesia or in the outpatient surgery settings. Our excision margins usually change according to the size, type, and location of the lesions. We use the minimal safety margins in areas like the periorbital region and send the tumor for frozen section examination during the operation to ensure complete removal of the tumor. For lesions less than 1 cm in diameter we use a 3 mm margin for excision. We increase the excision margin 1 mm for every increase of 1 cm in tumor diameter. We use a safety margin of 4 mm for morpheaform BCC and 5 mm for recurrent BCC. The safety margins may well be increased according to the degree of destruction of tissues in recurrent BCCs. In areas where there is no delicate structure nearby such as the back region, we prefer to use a wider excision margin in our excision spectrum. It is certainly better not to leave any residual tumor after the first operation as long as the surgical result is not compromising the aesthetic result. Frozen section examination or Mohs surgery may be used anywhere any suspicion about the completeness of removal arises. The wound may be left for secondary healing, closed primarily, skin grafted, or closed using a flap. The specimen is sent to the pathology laboratory with results transmitted within a few days. The final surgical decision is made on the basis of these results. A flap is used only after the final margins are negative. In cases where pathologic examination shows involved resected margins, we prefer reexcision because reported recurrence rates for incompletely excised basal cell carcinomas can be as high as 86% [13].In our series 6% of the patients required reexcision because of involved margins. Forty-three percent were in the nose, 25% were in the periauricular area, and 19% were in the periorbital area. 

Mohs micrographic surgery aims to completely remove the tumor via consecutive excision of the tumor, spatially orienting the specimen, histologically examining the margins, re-excising the residual tumor, and repeating the cycle until the area is tumor free. It is based on the principle that the tumor spreads by contiguous growth. The cure rates for primary BCCs <2 cm treated with MMS approach 99% [14, 15]. Recurrent BCC cure rates range from 94 to 96% [14, 15]. Mohs micrographic surgery is indicated for the treatment of recurrent BCC, primary BCC occurring at sites with high rates of recurrence (e.g., periorbital, Periauricular, and nasolabial areas), histologically difficult BCC (i.e., morphealike), and BCCs in which conservation of tissue is critical (e.g., on the nose and ear).

Destructive methods like electrodesiccation, curettage, cryosurgery and laser are appropriate methods for the management of smaller lesions that have low recurrence rates. Due to unacceptably high recurrence rates, poor cosmetic outcomes, and lack of histological control, it is generally not accepted as a first-line therapy for BCC in our center.

BCCs are sensitive to radiation. RT of tumors <2 mm has a cure rate of 90% for BCC [16]. However, larger lesions have a much lower success rate. We reserve radiation for elderly patients who are poor surgical candidates or for patients having residual or recurrent tumors.

Topical treatment using 5-fluorouracil may be used to BCC. Because the penetration of the agent is limited, its use should be confined to superficial lesions. It is not in our routine. 

**Figure 9:**
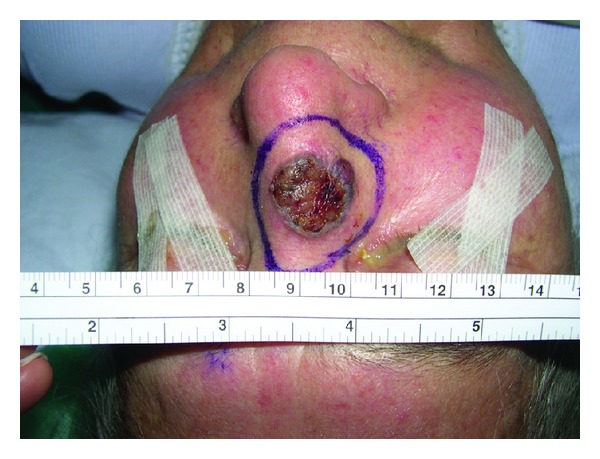
Recurrent BCC in the nose.

Although the results of primary excision are excellent, recurrences can occur. Recurrence rates are higher in the inner canthus, base of the nostril and preauricular, and postauricular areas [17]. This can be attributed to the scarcity of tissue, proximity to vital structures, and cosmetic considerations that must be taken into account in treating lesions on these locations. Recurrence rates are also increasing with increasing lesion size. We observed recurrence in 16 cases (3.08%). All occurred during the first four years. 8 were in the nose (Figure 9), 4 were in the periauricular area, and 4 were in the periorbital area. 

The incidence of incomplete excision of BCC reported in retrospective studies is in the range of 6.3 to 25 percent [18–27]. In our series, 12 BCCs (2.3%) required reexcision because of involved margins.

Although basal cell carcinoma is a malignant neoplasm, it rarely metastasizes. The rate of metastasis is below 0.1% [28]. This low rate can be explained by BCCs connective tissue stroma-dependent growth. Experimentally transplanted BCCs will not survive without dermal tissue [29]. Size, depth of invasion, and histological type are important predictors for metastasis [30]. Favored sites of metastasis include regional lymph nodes, liver, lung, bone, and skin. This rare metastasis is twice as common in males as in females.

## 5. Conclusion

Although there is relatively low attributable mortality, the morbidity and cost of treatment are significant. A large body of information serves as a foundation for oncologic principles, diagnosis methods, surgical excisions, follow-up protocols, and reconstructive methodologies that are currently in use. Surgical ablation remains the mainstay of treatment.
